# Uptake of Retrograde Tracers by Intact Optic Nerve Axons: A New Way to Label Retinal Ganglion Cells

**DOI:** 10.1371/journal.pone.0128718

**Published:** 2015-06-11

**Authors:** Yu-Xiang Liang, Jian Yang, Ti-Fei Yuan, Kwok-Fai So

**Affiliations:** 1 State Key Laboratory of Brain and Cognitive Sciences, the University of Hong Kong, Hong Kong, China; 2 Department of Anatomy, Li Ka Shing Faculty of Medicine, The University of Hong Kong, Hong Kong, China; 3 Department of Ophthalmology, the University of Hong Kong, Hong Kong, China; 4 School of Psychology, Nanjing Normal University, Nanjing, China; 5 GHM Institute of CNS Regeneration, Jinan University, Guangzhou, China; Hanson Institute, AUSTRALIA

## Abstract

Retrograde labelling of retinal ganglion cells with optic nerve transection often leads to degeneration of ganglion cells in prolonged experiments. Here we report that an intact optic nerve could uptake retrograde tracers applied onto the surface of the nerve, leading to high efficiency labelling of ganglion cells in the retina with long-term survival of cells. This method labelled a similar number of ganglion cells (2289±174 at 2 days) as the retrograde labeling technique from the superior colliculus (2250±94) or optic nerve stump (2279±114) after transection. This finding provides an alternative way to label retinal ganglion cells without damaging the optic tract. This will facilitate anatomical studies in identifying the morphology and connectivity of retinal ganglion cells, allowing secondary or triple labelling manipulations for long-term investigations.

## Introduction

Retrograde neuroanatomical tracing of retinal ganglion cells (RGCs) can specifically label these neurons which located in the innermost layer of the retina [1, 2], allowing the accurate evaluation of quantitative and morphological changes of these cells [3]. The superior colliculus (SC) labelling approach is currently the most common method to label RGCs [4–7]. In rats and mice, 98% of RGCs project to the contra-lateral SC, their main retino-recipient area in the brain [7–11]. Most RGCs can therefore be retrogradely labelled with fluorescent dyes applied onto the surface of the SC after removing the covering cortex. This is achieved through opening the skull and removing the underlying cortex using a needle connected to a vacuum pump. After the bleeding has stopped, a piece of gel foam soaked with retrograde tracer is placed on the surface of the SC [12, 13]. However, the removal of large cortical areas leads to injury to the brain, with neuroinflammation lasting for several days after the procedure. It is also possible to inject the retrograde tracers into the SC through a micropipette [14], causing minimal damage to the cortex; however, this sometimes appears to be insufficient to label all RGCs if the technique was not mastered skillfully.

Optic nerve (ON) stump labelling is employed when the SC labelling pathway is disrupted, such as in an ON cut or ON crush animal model. This approach requires a complete transection of the ON several millimeters behind the optic disc, and subsequent adherence of a small piece of gel foam (absorbable gelatin) soaked in retrograde tracers to the ON stump [15, 16]. This approach can lead to complete labelling of all RGCs, but the axotomy will inevitably lead to the death of RGCs; in adult mammals, severing of RGC axons in the ON leads to the death of the axotomized neurons from several days until months [17–21].

In previous practices, we occasionally found that RGCs could uptake and be labelled by retrograde labelling dyes applied onto the surface of the intact ON, suggesting for a possible way to label most of the RGCs without damaging the optic tract. To test our hypothesis, we applied two different fluorescent tracers onto the intact ON in order to label the RGCs, before completing quantitative studies of efficacy. Interestingly, we found that this approach led to consistent labelling of RGCs within several days; at early time points, the labeling efficiency seems comparable to the SC method and ON stump method.

## Methods

### Ethics Statement

The use of animals followed the requirements of the Cap. 340 Animals (Control of Experiments) Ordinance and Regulations in Hong Kong. All the experimental and animal handling procedures were approved by the University of Hong Kong Animal Ethics Committee (committee of use of laboratory animals for teaching and research, CULATR, CULATR # 1937–09).

### Animals

38 adult female Sprague–Dawley (SD) rats, weighing approximately 250 g, aged 8–10 weeks, were used in the experiments. The animals were housed with free access to food and water under a 12-hour light/12-hour dark cycle (7:00 AM–7:00 PM). During all surgical operations, the animals were anesthetized and maintained with muscular injections of a mixture of ketamine (80 mg/kg) and xylazine (8 mg/kg). For optic stump and intact ON retrograde labelling, 0.5% alcaine (Alcon-Couvreur, Puurs, Belgium) was applied to the eyes prior to the surgery, and antiseptic eye drops (Tobres [Tobramycin 0.3%]; lcon-Couvreur) were used to prevent infection after the procedures. Finadyne (0.025 mg/mL) in drinking water was applied for 7 days after surgery to relieve the pain when needed. All animals were sacrificed with overdose of pentobarbital at different time points.

35 out of the 38 animals were subjected for RGC filling experiment, 6 were used for bilateral SC labelling (sacrificed 7 days later), 10 for bilateral intact ON labelling (Left Fluoro-Gold-FG, Right Granular Blue- GB, 3 sacrificed 2 days later, 3 at 7 days later, 2 at 2 weeks, 2 at 3 weeks), 12 for unilateral intact ON labelling (FG,3 sacrificed at 2days, 3 sacrificed at 7 days later, 4 at 2 weeks later, and 2 at 3 weeks later) with contralateral optic stump labelling (FG), and 7 for unilateral optic stump labelling (FG, 3 at 2 days, 2 at 7 days, 2 at 2 weeks later).

For histological verification of the axonal integrity on semi-thin resin sections (3 animals), 1 animal was left intact, 2 animals were subjected to bilateral intact ON labeling (1 scarified on 4 days, and 1 on 7 days).

### SC labelling

Post animal sedation, the SC was exposed bilaterally after removal of a small piece of skull with the underlying meninges and parts of the cortex and hippocampus covering the SC. After the bleeding was stopped, a piece of gel foam (Pharmacia & Upjohn, New York, NY) soaked with Fluoro-Gold (FG, 6% in distilled H_2_O; Fluorochrome, Denver, CO, USA) was placed on the surface of the SC and left there, then the overlying skin was closed and sutured. The animals were sacrificed 7 days later and the retinas were harvested and whole-mounted for cell counting.

### ON stump labelling

For ON stump labelling, the animal was sedated and then the posterior pole of the left eye was exposed through a superior temporal intra-orbital approach. The left eyelid was lifted up using a suture, and the bulbar conjunctiva was cut coronally to expose the superior extraocular muscles. By lifting up the muscles using forceps, the intraorbital portion of the ON was exposed and its dura sheath was opened longitudinally. A complete transection was made to the ON 1.5 mm posterior to the optic disc, as previously described [22]. A piece of gel foam soaked with 6% FG was placed at the proximal optic stump and left there. Care was taken to maintain the blood supply to the retina. The animals were sacrificed 2, 7, 14, and 21 days later, and retinas were harvested and whole-mounted for cell counting.

### Intact ON labelling

For intact ON labelling, the intra-orbital portion of the ON was exposed as described in the optic stump model, the tissue around the ON was later cleaned. A piece of gel foam soaked with 6% FG was placed on the top and bilateral sides of the ON for better coverage and contact with the entire circumference of the ON. The gel foam was left there with the dura sheath maintained intact without opening. The animals were sacrificed 2, 7, 14, and 21 days later, and retinas were harvested and whole-mounted for cell counting. For Granular Blue (GB, Sigma, Germany) labeling, the procedures were performed as for FG labeling.

### Immunohistochemistry

Retinal whole mount immunohistochemistry was performed as previously described [23]. Briefly, the whole retina was immersed in 24-well plate (Sigma, HK) containing 200 μl 0.3% triton, 5% goat serum (sigma, HK)-PBS for 1 hour. Then the retina was incubated with rabbit-anti Iba1 (1:500, Wako, Japan) and mouse anti-beta-tubulin (1:200, Abcam, HK) for 24 hours at 4 degree. Then the retina was incubated with goat-anti rabbit 488 and goat-anti-mouse 568 (1:200, Invitrogen, HK) for 2 hours at room temperature for secondary antibody binding. Finally the retina was flat mounted on Dako (US) precoated slides for confocal imaging (Zeiss LSM 710, Germany).

### Semi-thin section with toluidine blue staining

The procedure was performed as previously described [24]. Retina was fixed in 2% PFA and 2.5% glutaraldehyde (Merck KGaA, Darmstadt, Germany), and prepared for semi-thin (0.5 μm) section on Lecia microtome (Germany). Then the semi-thin sections were stained with 1% toluidine blue (Bio-Rad, Life Science Research, Hercules, CA, US) for 1 minute, followed by section dehydration and mounting before visualization under light microscope (Zeiss, Germany).

### Quantification of RGCs

The quantification of RGCs in the retrogradely labelled retina was performed as previously described [25, 26]. Briefly, at the predefined time points, animals were sacrificed with an overdose of pentobarbital. After transcardial perfusion with 0.9% saline, the eyes were enucleated and fixed in 4% paraformaldehyde for two hours. The retinas were then dissected, washed in 0.1M Phosphate Buffer (PB) and flat-mounted onto slides. The slides were examined under a Zeiss microscope. Pictures of FG labelled RGCs consisting of an area of 200 μm x 200 μm were taken under x 40 objective for subsequent RGC counting using an ultraviolet filter (excitation wavelength 330–380 nm), along the midline of each quadrant, starting from the point at 400 μm away from the optic disc and moving to the border at 500 μm intervals. Four quadrants, each divided into eight microscopic fields for a total of about 32 fields per retina, were photographed. RGC quantification was performed by manually counting the RGC number in the photographs on a computer screen. Data of FG-labelled RGCs/mm^2^ were expressed in terms of “mean±SEM”

### Statistics

The data were expressed in terms of “mean±SEM” and analyzed with SPSS 15.0 software (Chicago, US). The differences between two groups were examined by t test, and analyses of variance and P<0.05 was considered as statistically significant. Analyses using data from multiple eyes were following the procedures as previously described [27–31].

## Results

### Intact ON labelling leads to high efficiency of retrograde RGC filling

**Fig 1:**
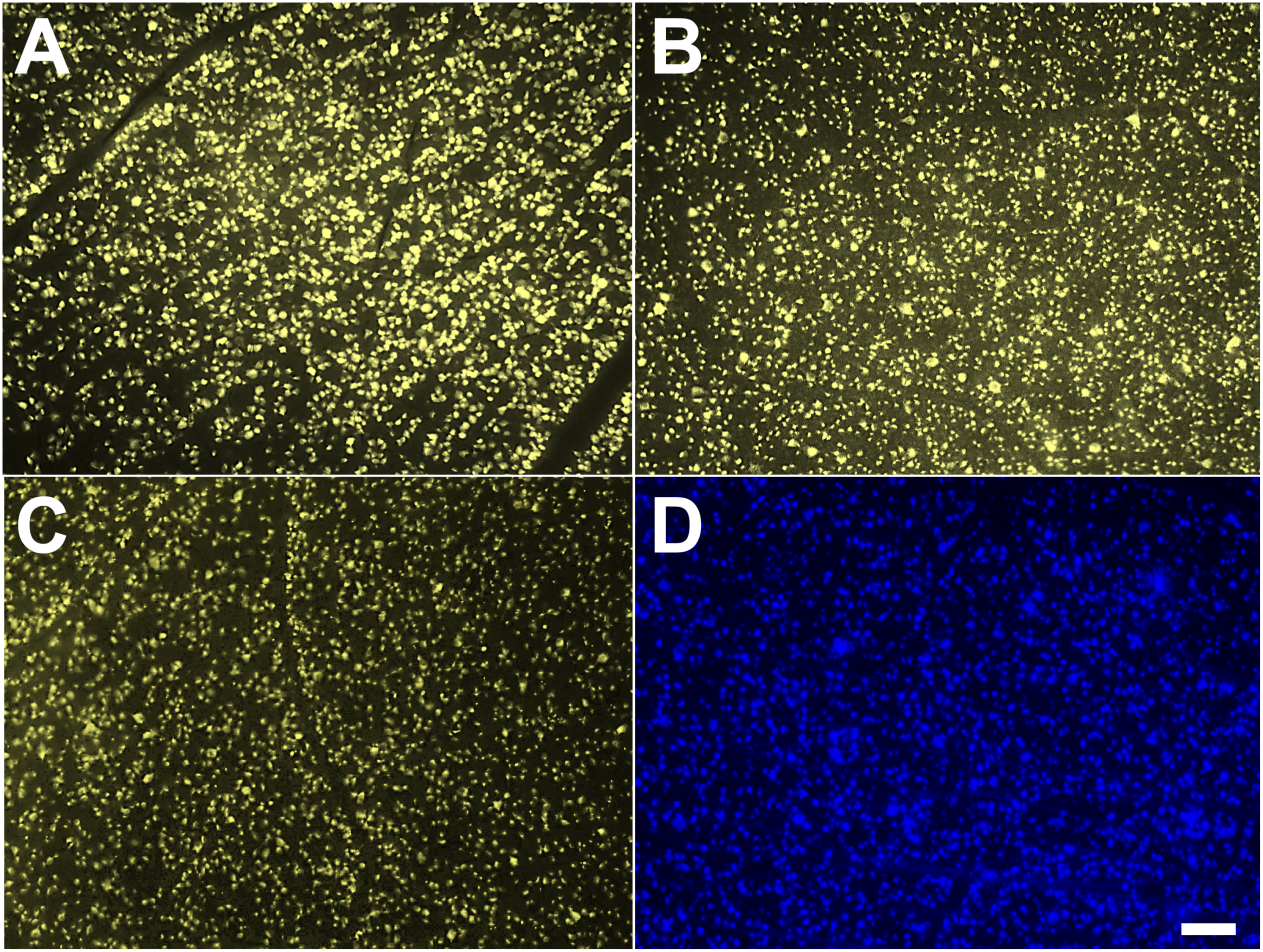
Fluoro-Gold (FG) application onto the surface of the optic nerve (ON) is sufficient to label all RGCs at 2 days. 1A: FG labelling of RGCs through superior colliculus application after 2 days; 1B: FG labelling of RGCs through ON cut approach after 2 days; 1C: FG labelling of RGCs through intact ON approach after 2 days; 1D: Granular Blue (GB) labelling of RGCs through intact ON approach after 2 days. Scale bar represents 100 μm.

**Fig 2:**
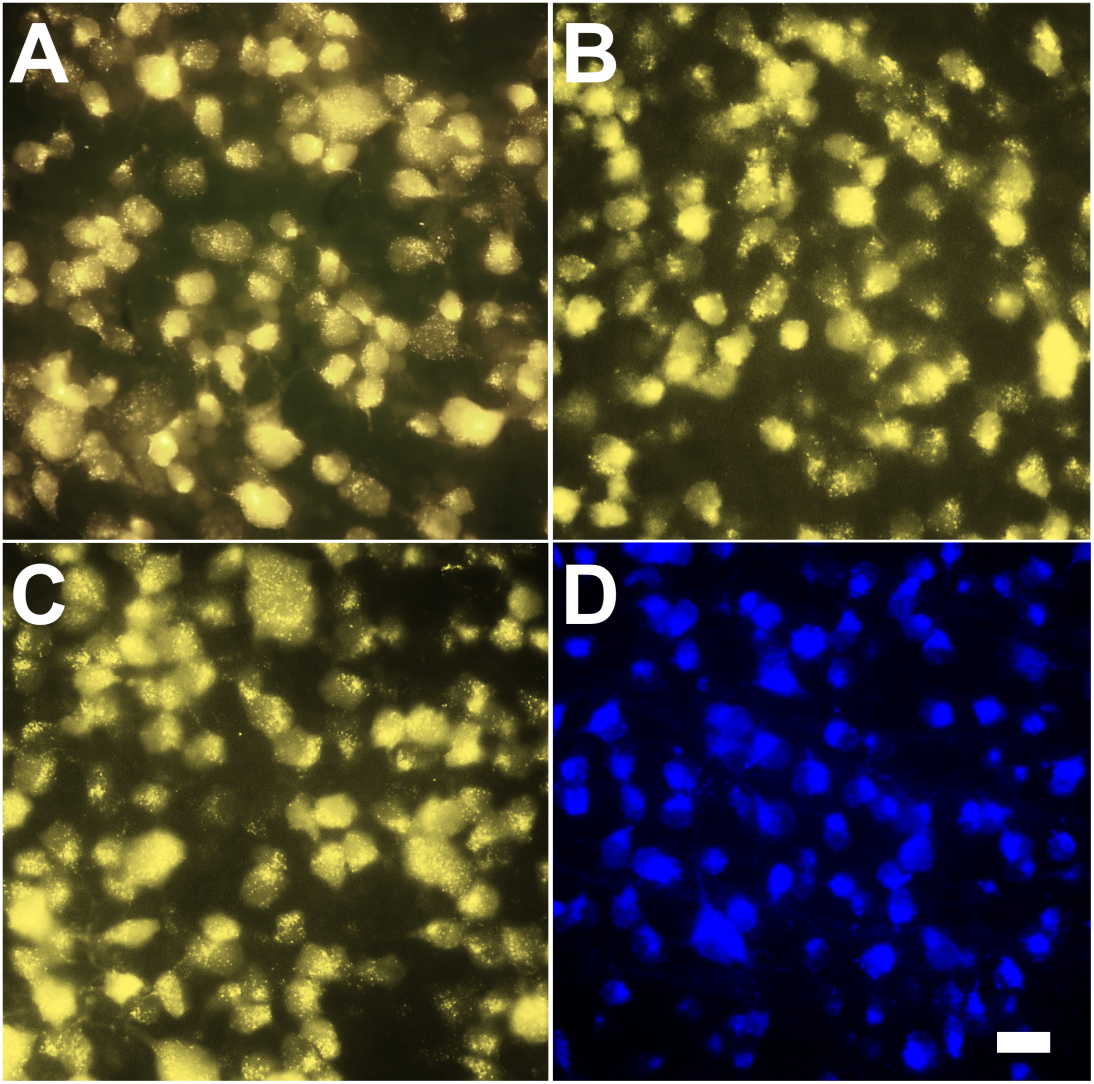
Intact ON labelling approach results in the same quality of RGC filling with fluorescent dyes. 2A: FG labelling of RGCs through superior colliculus application; 2B: FG labelling of RGCs through ON cut approach; 2C: FG labelling of RGCs through intact ON approach; 2D: GB labelling of RGCs through intact ON approach. Scale bar represents 20 μm.

We found that in our study, SC labelling and ON stump labelling are able to label most RGCs in the retina, as previously reported. Surprisingly, applying FG onto the surface of the ON was also sufficient for labelling of RGCs after 2 days of labelling (Fig 1), with the same levels of fluorescence intensity and soma filling of the RGCs (Fig 2). The intensity of fluorescence met the routine requirements for following cell counting and morphological studies. We also quantified the RGCs number per mm^2^ with different labelling methods (Table 1). At 2 days, the labeling efficacy of intact ON labeling was comparable to SC labeling and optic nerve stump labeling.

**Table 1:** RGCs numbers per mm^2^ using different labelling methods.

Group	SC (FG) 7d	ON stump (FG) 2d	Intact ON (FG) 2d	Intact ON (FG) 7d	Intact ON (GB) 2d
Mean±SEM (RGCs/mm2)	2250±94	2279±114 ^#^	2289±174 ^#^	1592±119 *	1509±145 *
n	6	6	6	6	4

Note:

^#^ for non-significant.

* For P<0.05 in compared to SC labeling.

### Intact ON labelling shows certain stability in prolonged time points

**Fig 3:**
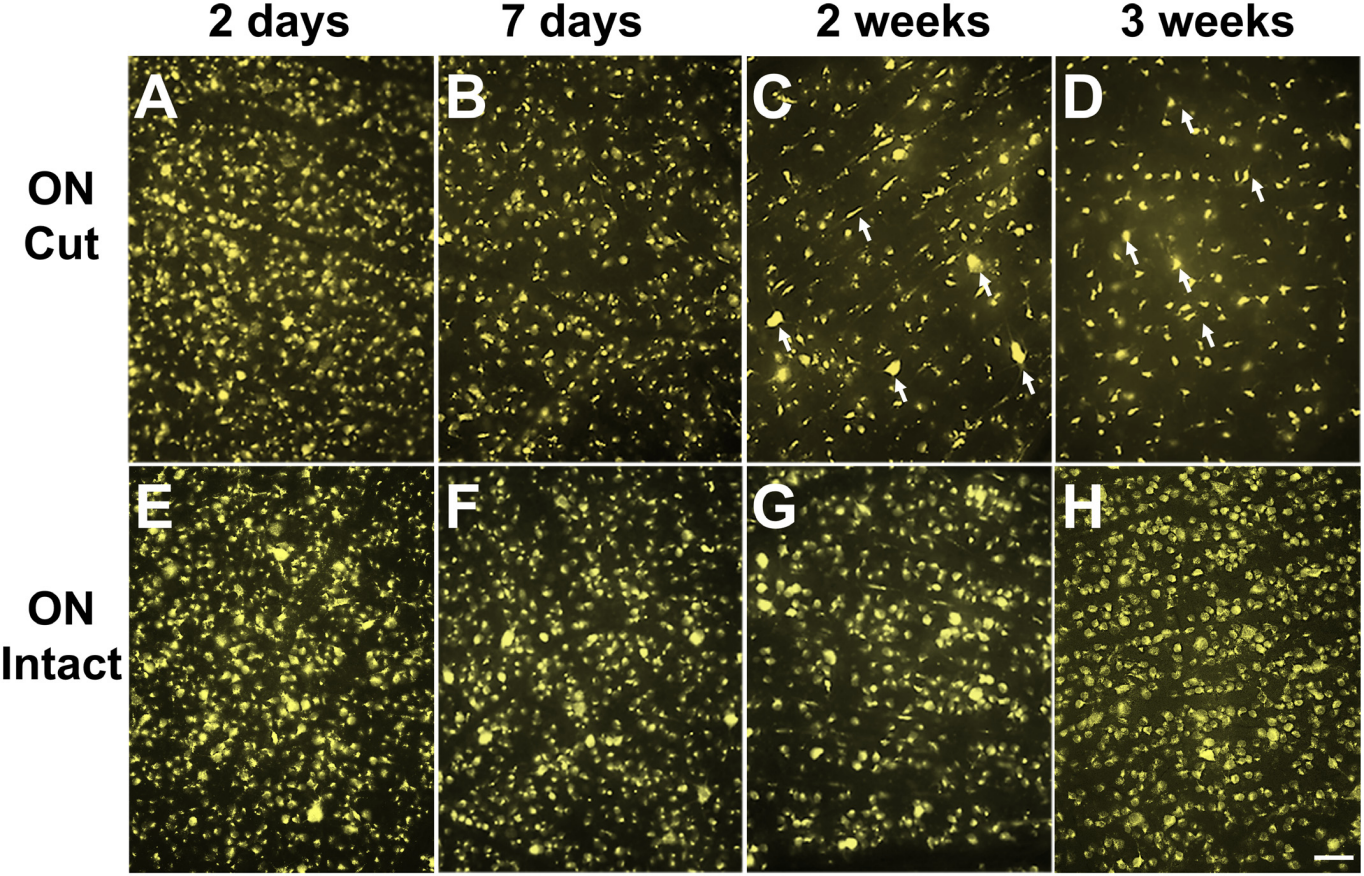
Intact ON labelling approach leads to stable filling without subsequent RGC loss. 3A, B, C, and D represent FG labelling of RGCs through ON cut approach at 2 days, 7 days, 2 weeks and 3 weeks. 3E, F, G, and H represent FG labelling of RGCs through intact ON approach at 2 days, 7 days, 2 weeks and 3 weeks, respectively. Arrows in C and D are showing residual RGCs that are with big soma size (potentially alpha-RGCs). Scale bar represents 50 μm.

The ON transection leads to gradual death of RGCs. Indeed, the density of labelled RGCs gradually declined as labelling time extended to 7 days, 2 weeks and 3 weeks in the ON stump group retinas. After 3 weeks, most RGCs had died and the RGC layer was mainly occupied by FG-phagocytizing microglia (Fig 3, white arrow). However, with intact ON labelling, though there were some FG-phagocytizing microglia, the density of RGCs remained high while the fluorescence signals were stable (Fig 3). It is noted that the number of labeled RGCs with intact ON approach slightly decreased (Table 1), possibly due to the degradation of FG out of the RGCs.

### Labelling dyes were taken-up by intact axons, not through penetration of sclera

**Fig 4:**
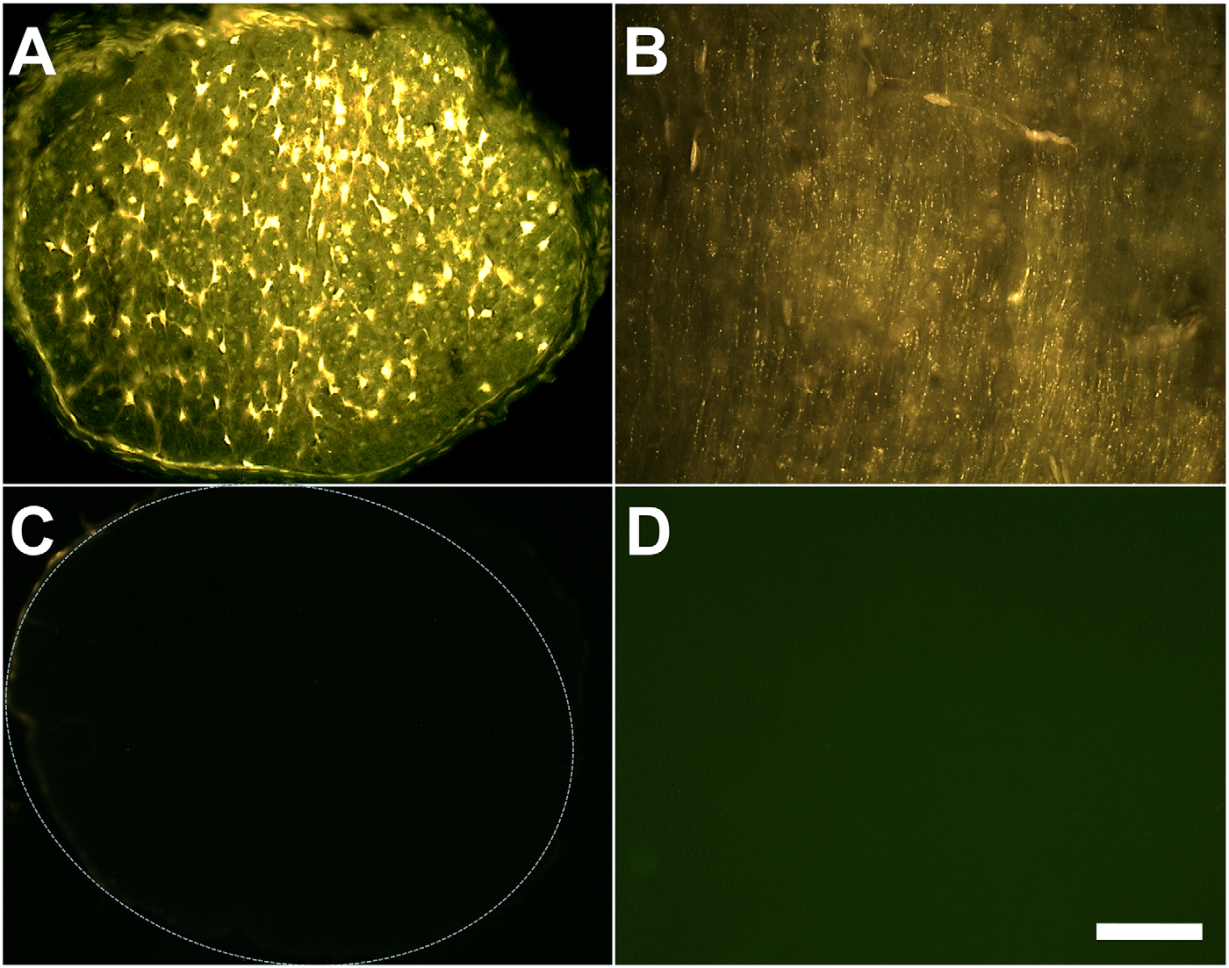
FG application onto the surface of ON could be taken up by intact ON fibers. 5A: Cross section of FG labelled ON; 5B: Longitudinal section of FG labelled ON; 5C: Cross section of ON through sclera approach; 5D: Picture of sclera approach labelled retina. Scale bar represents 50 μm.

To confirm that the RGCs were labeled by intact axons’ directly taking up the retrograde labelling dye, we collected the ON from the FG intact ON labelled group for cryo-section and examined the sections. We found that intact ON fibers could indeed be labeled as a result of taking up FG (Fig 4). To further exclude the possibility that the RGCs might have been labelled by dyes that penetrated the sclera, we applied FG/GB on the outer wall of the eye ball far away from the ON. With this approach of dye application, neither the ON nor any RGCs were labelled (Fig 4).

### Intact ON labelling is suitable for other retrograde tracers

**Fig 5:**
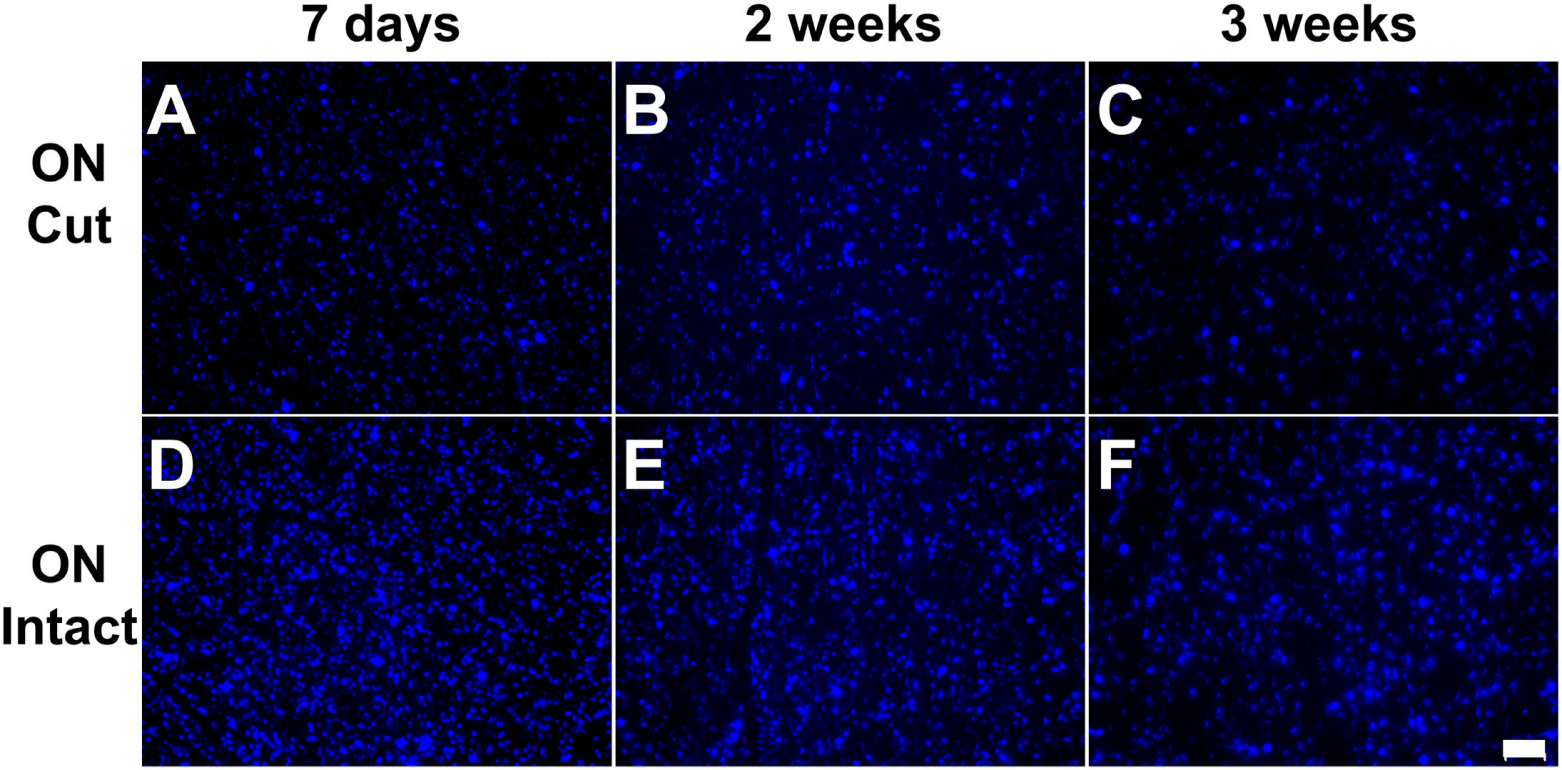
Intact ON labelling approach is also feasible for GB filling of RGCs, with increased stability compared to ON cut approach. 4A, B, and C represent ON cut approach with GB labelling at 7 days, 2 weeks and 3 weeks; 4 D, E, and F represent intact ON approach with GB labelling at 7 days, 2 weeks and 3 weeks, respectively. Scale bar represents 50 μm.

In addition to FG, we found that GB could also act as a retrograde tracer for intact ON labelling, with slightly lower efficacy (Fig 5). We believe that this approach is applicable to different retrograde tracers.

### Decreased FG labeling without RGC loss at prolonged time points

**Fig 6:**
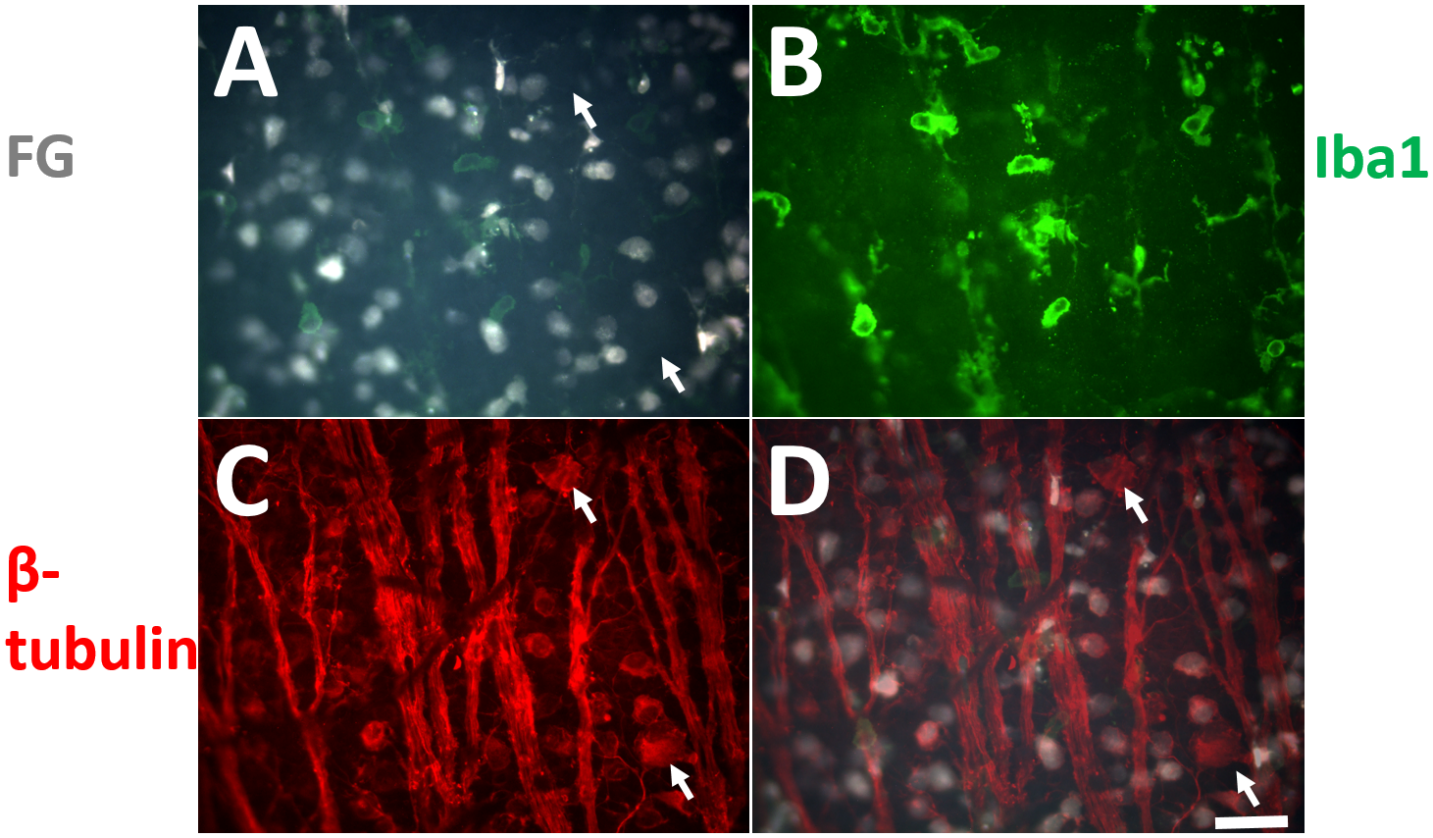
Decreased FG labeling without RGC loss at prolonged time points. A: FG labeling with intact ON approach at 7 days. B: IBA1 immunohistochemistry showing microglial cells. C: beta-tubulin immunohistochemistry showing all RGCs and axons. D: merged image of A and C. Arrows in C and D are showing RGCs without FG filling. Scale bar represents 50 μm.

We asked if the decreased number of labeled RGCs is due to the cell death after intact ON labeling. With double immunohistochemistry of beta-tubulin and IBA1 (microglia marker), we found FG-unlabeled RGCs at 7 days after intact ON labeling, suggesting that the FG might be removed from the labeled RGCs (Fig 6). In addition, there were few microglial cells with FG labeling and slight activation, suggesting that the microglial cells might be responsible for removing excess FG molecules in retina.

### Intact ON labeling results in minimal injury to optic nerve

**Fig 7:**
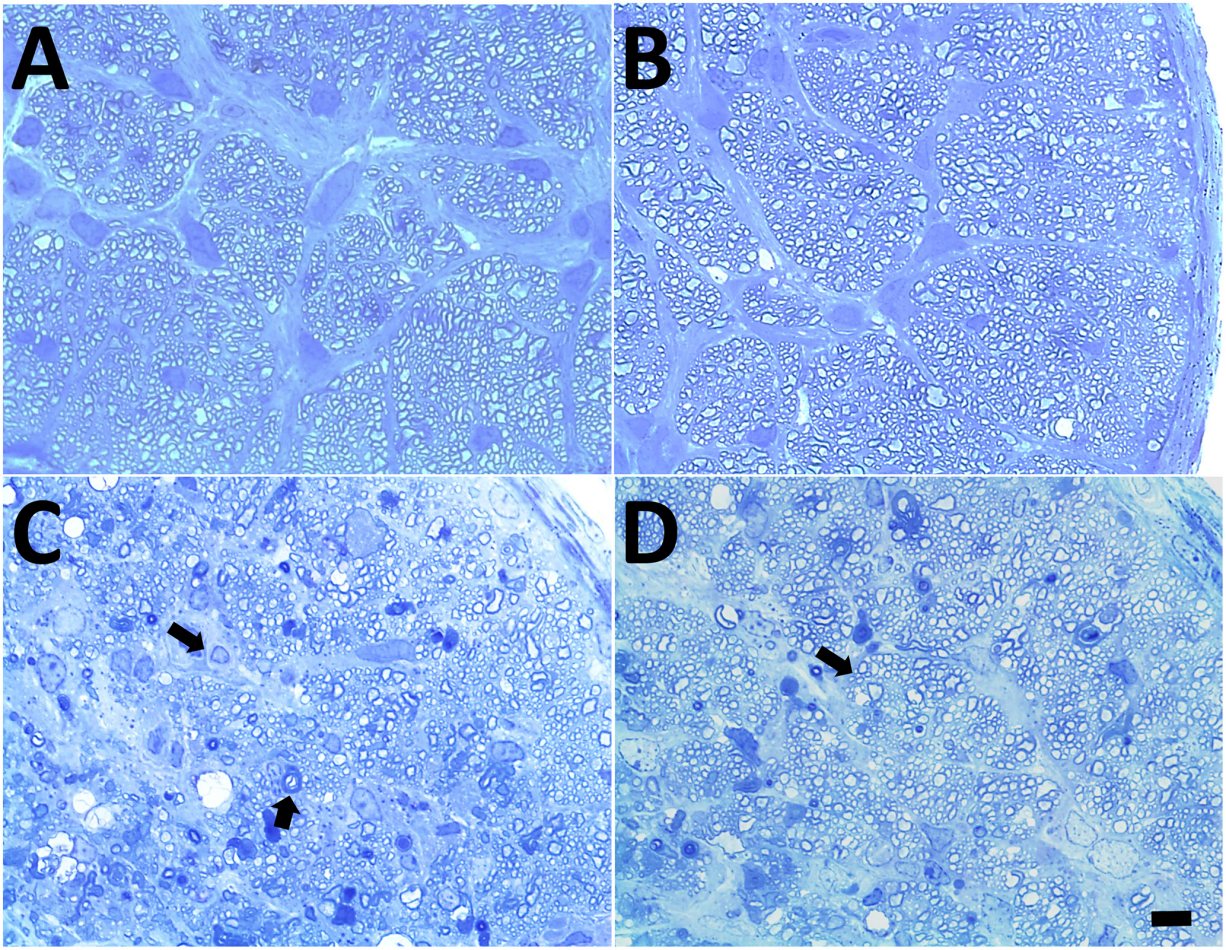
Intact ON labeling results in minimal injury to ON. 7A, B: normal ON. 7C: 4 days after intact labeling. 7D: 7 days after intact labeling. Arrows in C and D indicate the myelin damage. Scale bar represents 10 μm.

We performed semi-thin (0.5 μm) toluidine blue section for axonal integrity investigation (Fig 7). In compared to normal ON (Fig 7A and 7B), ON following intact labeling approach exhibited minimal, if any, disruption of integrity (arrows) at 4 days (Fig 7C) and 7 days (Fig 7D) after manipulation. However, the extent of axonal damage still requires quantitative investigations in the future.

## Discussion

Retrograde transportation conveys molecules/organelles away from the synapse or plasma membrane towards the cell body or soma. The dynein-mediated retrograde transport is active during certain cellular processes, such as delivering chemical messengers or endocytosis products bound for endolysosomes from the axon to cell body. In the early 1970s, the employment of axonal-transported tracers revolutionized the field of neuroanatomical tracing relative to the previously dominant reduced silver staining approach [32–34]. In the following decades, axonal tracing of neuronal pathways *via* anterograde or retrograde transport became one of the most important approaches for researchers to label newly born, transplanted, damaged or axotomized neurons under various conditions, such as central nervous system (CNS) lesion or neurodegenerative diseases. Specifically in the field of Ophthalmology, retrograde axonal tracing has become an important and major approach for quantitative studies investigating the survival or degeneration of RGCs [35].

### Retrograde tracers

Retrograde tracers are typically dyes unconjugated or conjugated with a fluorescent probe or neuronal fluorescence tracers, such as Evans Blue, GB, Fast Blue, and FG since neurons labelled by these tracers are easily detected under common fluorescence microscopes. In addition, Horseradish Peroxidase (HRP), Wheat germ agglutinin (WGA), Cholera toxin-B chain (CTB), and Tetanus toxin-fragment C (TTC) are often used for neuronal tracing. Detection of these retrograde tracers often requires chemical or immunohistochemical reactions in fixed brain tissues. In recent decades, however, different viruses such as Herpes Simplex Virus, Adeno Virus, and Pseudo rabies viruses, have been employed for retrograde neuronal tracing, taking advantage of their spreading pathways in nature. Since their introduction, genetic engineering of these viruses has led to their reduced toxicity and combined expression of different fluorescent proteins [36].

### FG and its uptake

FG, a fluorescent retrograde marker applicable for neuroanatomical tracing, is a tracer of choice for many laboratories and is often used in labelling RGCs [4, 5, 15]. The molecular weight of FG is 532.6 Daltons. It has been sold by Fluorochrome and widely used since 1985. It is reported that FG can be taken up through axon terminals or injured axons of neurons and retrogradely transported to cell bodies. By these means, FG is capable of labelling the neurons projecting to its specific region of application. Compared to retrograde markers, FG presents several advantages. Neurons labelled by FG can be visualized directly without additional processing. FG is capable of labelling distal dendrites extensively, after application FG is relatively stable for longer periods during different fixation processes and under various histochemical conditions.

Previous studies reported that FG can only be taken up by axon terminals and injured axons, but not intact axons at non-terminal sites (“axons of passage”) [37, 38]. However, our results showed that FG can be taken up by intact and myelinated axons of RGCs. The mechanism of intact ON labelling by FG is not clear. It has been reported that hydroxystilbamidine, chemically a weak base, is the active constituent of FG. In this respect, similar to True Blue, DAPI (4’, 6-Diamidino-2-phenylindole), GB, Bis-benzimide, Nuclear Yellow and several other retrograde transported molecules, FG should be able to cross cell membranes in its uncharged form. Furthermore, with a favorable pH gradient, FG should become trapped in bases in acidic cellular compartments *via* a well-understood process that can be found throughout many biological systems [39]. In this study, we tested this hypothesis with another retrograde tracer, GB (Fig 1D), which has a Molecular Weight of 486.3936 g/mol and forms a suspension in 0.9% saline solution. The results showed that, similar to FG, GB could also be taken up by intact ON fibers. Therefore, the application of retrograde tracers might interfere with passage fibers at the application site, causing unwanted labelling of different pathways. How severe the contamination would occur in the brain, however, is yet to be tested.

### The feasibility of labelling RGCs by intact ON

Though SC labelling is regarded as the standard way to access the population of RGCs in the retina, the technique is difficult to master and can cause damage to the brain. ON stump labelling as an alternative method of SC labelling also causes significant damage to RGCs because the axons are transected. These methods also limit the flexibility of experimental designs that require long-term survival of RGCs, or visual behavior experiments. The findings that intact ON fibers can uptake retrograde dyes leads to a novel “non-invasive” approach for RGC labelling, with minimum injury to the animal. The immediate filling of RGC allows cell quantification, morphological study, and, possibly, multiple rounds of cell labelling.

It is however noted that the filling quality with the intact ON approach is less “complete” as compared to the SC and ON stump approaches; this is potentially due to the number of FG molecules available for intact optic nerve axons. At prolonged time points, we found RGCs that were not FG-positive, such as after 7 days. Therefore, the current intact ON approach with FG is most suitable for experiments requiring immediate RGC filling with maintenance of optic tract integrity (Table 2). In the future, it will be interesting to employ fluorescence tracers with long-term stability in RGCs for persistent labeling studies [40]. Notably, there are signs of axonal injury with intact ON approach as well. However, the extent of axonal damage still requires quantitative investigations in the future.

**Table 2:** Summary of the three approaches.

	Labeling efficiency				
	2 days	7 days	14 days	RGC loss	RGC filling quality
SC	++	++++	+++++	/	+++++
ON stump	+++++	++++	+	+++++	+++++
Intact ON	+++++	++++	+++	/	+++

## Conclusion

In combination with certain retrograde tracers, we showed that intact ON fibers in the optic tract could uptake labelling dyes and retrogradely transport them to RGC cell bodies, thus leading to reliable RGC labelling. This intact ON labelling technique is a feasible way to label the whole population of RGCs, with labelling efficacy comparable to SC labelling and ON stump labelling approaches. Intact ON labelling provides an efficient way to label RGCs without causing severe damage to the brain or ON of the animal.

## References

[1] Thanos S, Vidal-Sanz M, Aguayo AJ. The use of rhodamine-B-isothiocyanate (RITC) as an anterograde and retrograde tracer in the adult rat visual system. Brain Res. 1987;406(1–2):317–21. . 2436717 10.1016/0006-8993(87)90799-2

[2] Vidal-Sanz M, Villegas-Perez MP, Bray GM, Aguayo AJ. Persistent retrograde labeling of adult rat retinal ganglion cells with the carbocyanine dye diI. Exp Neurol. 1988;102(1):92–101. . 3181354 10.1016/0014-4886(88)90081-7

[3] Galindo-Romero C, Aviles-Trigueros M, Jimenez-Lopez M, Valiente-Soriano FJ, Salinas-Navarro M, Nadal-Nicolas F, et al Axotomy-induced retinal ganglion cell death in adult mice: quantitative and topographic time course analyses. Exp Eye Res. 2011;92(5):377–87. 10.1016/j.exer.2011.02.008 . 21354138

[4] Peinado-Ramon P, Salvador M, Villegas-Perez MP, Vidal-Sanz M. Effects of axotomy and intraocular administration of NT-4, NT-3, and brain-derived neurotrophic factor on the survival of adult rat retinal ganglion cells. A quantitative in vivo study. Invest Ophthalmol Vis Sci. 1996;37(4):489–500. . 8595949

[5] Danias J, Shen F, Kavalarakis M, Chen B, Goldblum D, Lee K, et al Characterization of retinal damage in the episcleral vein cauterization rat glaucoma model. Exp Eye Res. 2006;82(2):219–28. 10.1016/j.exer.2005.06.013 16109406 PMC1401487

[6] Reichstein D, Ren L, Filippopoulos T, Mittag T, Danias J. Apoptotic retinal ganglion cell death in the DBA/2 mouse model of glaucoma. Exp Eye Res. 2007;84(1):13–21. 10.1016/j.exer.2006.08.009 . 17074320

[7] Salinas-Navarro M, Mayor-Torroglosa S, Jimenez-Lopez M, Aviles-Trigueros M, Holmes TM, Lund RD, et al A computerized analysis of the entire retinal ganglion cell population and its spatial distribution in adult rats. Vision Res. 2009;49(1):115–26. 10.1016/j.visres.2008.09.029 . 18952118

[8] Lund RD. Uncrossed Visual Pathways of Hooded and Albino Rats. Science. 1965;149(3691):1506–7. 10.1126/science.149.3691.1506 . 17791642

[9] Perry VH. Evidence for an amacrine cell system in the ganglion cell layer of the rat retina. Neuroscience. 1981;6(5):931–44. . 6165929 10.1016/0306-4522(81)90174-3

[10] Linden R, Perry VH. Massive retinotectal projection in rats. Brain Res. 1983;272(1):145–9. . 6616190 10.1016/0006-8993(83)90371-2

[11] Salinas-Navarro M, Jimenez-Lopez M, Valiente-Soriano FJ, Alarcon-Martinez L, Aviles-Trigueros M, Mayor S, et al Retinal ganglion cell population in adult albino and pigmented mice: a computerized analysis of the entire population and its spatial distribution. Vision Res. 2009;49(6):637–47. 10.1016/j.visres.2009.01.010 . 19948111

[12] Danias J, Shen F, Goldblum D, Chen B, Ramos-Esteban J, Podos SM, et al Cytoarchitecture of the retinal ganglion cells in the rat. Invest Ophthalmol Vis Sci. 2002;43(3):587–94. . 11867571

[13] Chiu K, Lau WM, Yeung SC, Chang RC, So KF. Retrograde labeling of retinal ganglion cells by application of fluoro-gold on the surface of superior colliculus. J Vis Exp. 2008;(16). 10.3791/819 19066544 PMC2583033

[14] Kelly JP, Gilbert CD. The projections of different morphological types of ganglion cells in the cat retina. J Comp Neurol. 1975;163(1):65–80. 10.1002/cne.901630105 . 1159111

[15] Fu QL, Hu B, Wu W, Pepinsky RB, Mi S, So KF. Blocking LINGO-1 function promotes retinal ganglion cell survival following ocular hypertension and optic nerve transection. Invest Ophthalmol Vis Sci. 2008;49(3):975–85. 10.1167/iovs.07-1199 . 18326721

[16] Lafuente Lopez-Herrera MP, Mayor-Torroglosa S, Miralles de Imperial J, Villegas-Perez MP, Vidal-Sanz M. Transient ischemia of the retina results in altered retrograde axoplasmic transport: neuroprotection with brimonidine. Exp Neurol. 2002;178(2):243–58. . 12504883 10.1006/exnr.2002.8043

[17] Grafstein B, Ingoglia NA. Intracranial transection of the optic nerve in adult mice: preliminary observations. Exp Neurol. 1982;76(2):318–30. . 7095057 10.1016/0014-4886(82)90212-6

[18] Richardson PM, Issa VM, Shemie S. Regeneration and retrograde degeneration of axons in the rat optic nerve. J Neurocytol. 1982;11(6):949–66. . 7153791 10.1007/BF01148310

[19] Misantone LJ, Gershenbaum M, Murray M. Viability of retinal ganglion cells after optic nerve crush in adult rats. J Neurocytol. 1984;13(3):449–65. . 6481407 10.1007/BF01148334

[20] Villegas-Perez MP, Vidal-Sanz M, Rasminsky M, Bray GM, Aguayo AJ. Rapid and protracted phases of retinal ganglion cell loss follow axotomy in the optic nerve of adult rats. J Neurobiol. 1993;24(1):23–36. 10.1002/neu.480240103 . 8419522

[21] Selles-Navarro I, Villegas-Perez MP, Salvador-Silva M, Ruiz-Gomez JM, Vidal-Sanz M. Retinal ganglion cell death after different transient periods of pressure-induced ischemia and survival intervals. A quantitative in vivo study. Invest Ophthalmol Vis Sci. 1996;37(10):2002–14. . 8814140

[22] Vidal-Sanz M, Bray GM, Villegas-Perez MP, Thanos S, Aguayo AJ. Axonal regeneration and synapse formation in the superior colliculus by retinal ganglion cells in the adult rat. J Neurosci. 1987;7(9):2894–909. . 3625278 10.1523/JNEUROSCI.07-09-02894.1987 PMC6569122

[23] Yuan TF, Liang YX, Peng B, Lin B, So KF. Local proliferation is the main source of rod microglia after optic nerve transection. Scientific Reports. 2015;In press. 10.1038/srep10788 PMC4649910 26035780

[24] Li HY, Ruan YW, Kau PW, Chiu K, Chang RC, Chan HH, et al Effect of Lycium barbarum (Wolfberry) on Alleviating Axonal Degeneration After Partial Optic Nerve Transection. Cell Transplant. 2015;24(3):403–17. 10.3727/096368915X686896 . 25622224

[25] Li H, Liang Y, Chiu K, Yuan Q, Lin B, Chang RC, et al Lycium barbarum (wolfberry) reduces secondary degeneration and oxidative stress, and inhibits JNK pathway in retina after partial optic nerve transection. PLoS One. 2013;8(7):e68881 10.1371/journal.pone.0068881 23894366 PMC3716882

[26] Fu QL, Li X, Yip HK, Shao Z, Wu W, Mi S, et al Combined effect of brain-derived neurotrophic factor and LINGO-1 fusion protein on long-term survival of retinal ganglion cells in chronic glaucoma. Neuroscience. 2009;162(2):375–82. 10.1016/j.neuroscience.2009.04.075 . 19422885

[27] Ray WA, O'Day DM. Statistical analysis of multi-eye data in ophthalmic research. Invest Ophthalmol Vis Sci. 1985;26(8):1186–8. . 4019113

[28] Fan Q, Teo YY, Saw SM. Application of advanced statistics in ophthalmology. Invest Ophthalmol Vis Sci. 2011;52(9):6059–65. 10.1167/iovs.10-7108 . 21807933

[29] Rosner B. Statistical methods in ophthalmology: an adjustment for the intraclass correlation between eyes. Biometrics. 1982;38(1):105–14. . 7082754

[30] Murdoch IE, Morris SS, Cousens SN. People and eyes: statistical approaches in ophthalmology. Br J Ophthalmol. 1998;82(8):971–3. 9828786 10.1136/bjo.82.8.971 PMC1722711

[31] Armstrong RA. Statistical guidelines for the analysis of data obtained from one or both eyes. Ophthalmic Physiol Opt. 2013;33(1):7–14. 10.1111/opo.12009 . 23252852

[32] Schmued L, Kyriakidis K, Heimer L. In vivo anterograde and retrograde axonal transport of the fluorescent rhodamine-dextran-amine, Fluoro-Ruby, within the CNS. Brain Res. 1990;526(1):127–34. . 1706635 10.1016/0006-8993(90)90258-d

[33] Bentivoglio M, Kuypers HG, Catsman-Berrevoets CE. Retrograde neuronal labeling by means of Bisbenzimide and Nuclear Yellow (Hoechst S 769121). Measures to prevent diffusion of the tracers out of retrogradely labeled neurons. Neurosci Lett. 1980;18(1):19–24. . 6189012 10.1016/0304-3940(80)90207-4

[34] Bentivoglio M, Kuypers HG, Catsman-Berrevoets CE, Loewe H, Dann O. Two new fluorescent retrograde neuronal tracers which are transported over long distances. Neurosci Lett. 1980;18(1):25–30. . 6189013 10.1016/0304-3940(80)90208-6

[35] Danias J, Lee KC, Zamora MF, Chen B, Shen F, Filippopoulos T, et al Quantitative analysis of retinal ganglion cell (RGC) loss in aging DBA/2NNia glaucomatous mice: comparison with RGC loss in aging C57/BL6 mice. Invest Ophthalmol Vis Sci. 2003;44(12):5151–62. . 14638711 10.1167/iovs.02-1101

[36] Kobbert C, Apps R, Bechmann I, Lanciego JL, Mey J, Thanos S. Current concepts in neuroanatomical tracing. Prog Neurobiol. 2000;62(4):327–51. . 10856608 10.1016/s0301-0082(00)00019-8

[37] Schmued LC, Fallon JH. Fluoro-Gold: a new fluorescent retrograde axonal tracer with numerous unique properties. Brain Res. 1986;377(1):147–54. . 2425899 10.1016/0006-8993(86)91199-6

[38] Silverman AJ, Witkin JW, Silverman RC, Gibson MJ. Modulation of gonadotropin-releasing hormone neuronal activity as evidenced by uptake of fluorogold from the vasculature. Synapse. 1990;6(2):154–60. 10.1002/syn.890060206 . 2237778

[39] Wessendorf MW. Fluoro-Gold: composition, and mechanism of uptake. Brain Res. 1991;553(1):135–48. . 1933270 10.1016/0006-8993(91)90241-m

[40] Gomez-Ramirez AM, Villegas-Perez MP, Miralles de Imperial J, Salvador-Silva M, Vidal-Sanz M. Effects of intramuscular injection of botulinum toxin and doxorubicin on the survival of abducens motoneurons. Invest Ophthalmol Vis Sci. 1999;40(2):414–24. . 9950601

